# Synergistic Response of Rifampicin with Hydroperoxides on *Mycobacterium*: A Mechanistic Study

**DOI:** 10.3389/fmicb.2017.02075

**Published:** 2017-10-31

**Authors:** Yesha S. Patel, Sarika Mehra

**Affiliations:** Department of Chemical Engineering, Indian Institute of Technology Bombay, Mumbai, India

**Keywords:** tuberculosis, synergism, rifampicin, cumene hydroperoxide, combination therapy, oxidative stress, lipid peroxidation, membrane permeabilization

## Abstract

Prolonged chemotherapy as well as rapid development of antimicrobial resistance are two of the major concerns for treatment of mycobacterial infections. To enhance the effectiveness of current drug regimens, search for compounds having synergistic interaction with anti-mycobacterial drugs has become indispensable. Here, we have investigated the intervention by oxidative stress, a major factor in mycobacterial pathogenesis, in combination with rifampicin (RIF), a first-line drug used against *Mycobacterium tuberculosis*. We have observed that a sub-inhibitory concentration of cumene hydroperoxide (CHP), a hydrophobic oxidant, synergistically reduced the minimum inhibitory concentration of RIF by fourfold, with a Fractional Inhibitory Concentration Index (FICI) of 0.45. Also, this interaction was found to be robust and synergistic against different strains of *M. smegmatis* as well as on *M. bovis* BCG, with FICI ranging from 0.3 to 0.6. Various physiological, biochemical and molecular parameters were explored to understand the mechanism of synergy. It was observed that increased membrane permeability owing to the presence of the oxidant, led to higher uptake of the drug. Moreover, downregulation of the hydroperoxide reductases by RIF, a transcriptional inhibitor, prevented quenching of the reactive oxygen species produced in the presence of CHP. The lipid soluble reactive species triggered autocatalytic lipid peroxidation (LPO), observed here as extensive membrane damage eventually leading to growth inhibition. Furthermore, it was seen that in combination with hydrogen peroxide (H_2_O_2_), the effect was only additive, establishing LPO as a key aspect leading toward synergism. To conclude, this work suggests that targeting the bacterial membrane by a radical species can have a significant impact on the treatment of tuberculosis.

## Introduction

Mycobacterial infections are responsible for millions of death worldwide every year, second only to the deaths caused by HIV (WHO, 2016). With the advent of antimicrobial resistance in recent times (Ormerod, 2005; Shah et al., 2007), the concern for treatment and eradication of these bacteria has increased significantly. Not only is the discovery of new antimicrobials severely lagging behind the rate of emergence of resistance (Singh, 2014), the probability of mycobacteria to develop resistance toward new drugs has also been observed to be very high. For example, Bedaquiline (Mahajan, 2013) and Delamanid (Xavier and Lakshmanan, 2014) were recently introduced in the anti-TB drug regime. However, in a short time-span resistance to both these drugs has emerged (Bloemberg et al., 2015).

To counter the deadly Mycobacteria and to prevent the development of resistance, tuberculosis drug regime comprises of a combination of multiple drugs (Mitchison, 2012; Kerantzas and Jacobs, 2017). With dwindling discovery of new drugs, combination therapy with existing non-tuberculosis drugs is being explored as an alternative strategy to identify novel drug pairs that have a synergistic effect on the bacteria (Rey-Jurado et al., 2013; Bruhn et al., 2015; Gonzaloa et al., 2015; Zhang et al., 2015; Zou et al., 2015). However, both the molecules in a combination do not need to have an antimicrobial effect. One of the components can be an adjuvant that does not have any antimicrobial property by itself, but enhances the activity of the drug such as efflux pump inhibitors (Pule et al., 2016) and β-lactamase inhibitors (Wright, 2016). In a recent work from our group, Poly Acrylic Acid (PAA) coated nanoparticles were shown to act as efflux pump inhibitors and enhance the efficacy of rifampicin against *Mycobacterium smegmatis* synergistically (Padwal et al., 2014). Similarly, citric-acid coated nanoparticles increased the permeability of cells thus making them more sensitive to rifampicin (Padwal et al., 2015).

A number of approaches have been employed to identify combination pairs that are synergistic in action against bacteria. High-throughput screening studies of antibiotics in combination with a large chemical library have been carried out to identify compounds as potential adjuvants (Ejim et al., 2011; Ramon-Garcia et al., 2011). In a screen against *M. tuberculosis*, bromperidol was identified to synergistically enhance the activity of several antibiotics. Other studies specifically targeted an essential function of the cell. For example, Farha et al. (2013) screened for compounds that selectively damage the proton-motive force in bacterial cells by disrupting the electrical potential or dissipating the transmembrane proton gradient in cells. In combination, these compounds were shown to act synergistically on *Staphylococcus aureus* (Farha et al., 2013). Sometimes these studies reveal novel molecules/combinations of drugs that can be used in treatment. Alternatively, understanding the mechanism of action of these compounds facilitates identification of proficient cellular targets to act upon and can lead to a rational design of combat strategies.

Reactive oxygen species (ROS) play an important role in cell death (Kashmiri and Mankar, 2014). Therefore, the oxidative stress pathway has been explored as an important drug target (Vatansever et al., 2013). On infection, oxidative burst is the foremost stress encountered by Mycobacteria (Kohchi et al., 2009). *In vitro* bacterial studies have illustrated that activity of isoniazid, a first line anti-Tb drug, increases in the presence of oxidative stress. This is primarily due to the upregulation of KatG, a catalase-peroxidase enzyme, by latter which is required to convert the INH prodrug into its active form (Bulatovic et al., 2002). Due to the importance of oxidative stress in the pathogenesis of mycobacteria and its synergistic response with INH, it represents a good candidate to be further investigated.

In the present study, we are evaluating the chemotherapeutic effect of oxidative stress in combination with rifampicin, another first-line anti Tb drug, on *M. smegmatis* as a surrogate model. Primarily, the impact of the combined treatment was estimated on the Minimum inhibitory concentration (MIC) of the bacteria. Furthermore, through exploring different physiological parameters we have elucidated the mechanism of synergy of this combination. To evaluate the robustness of this inhibitory role of oxidative stress, the effect was also verified on *M. bovis* BCG and strains of different genetic background of *M. smegmatis* having varying susceptibility profile toward the drug and/or oxidative stress.

## Materials and Methods

### Bacterial Strains and Culture Conditions

Wild-type *M. smegmatis* mc^2^155 (WT), obtained from All India Institute of Medical Sciences, Delhi (Taneja and Tyagi, 2007) was used for all experimental studies. For liquid culture, cells were grown in Middlebrook 7H9 broth (Himedia) supplemented with 0.44% glycerol (Sigma–Aldrich) and 0.15% Tween-80 (Sigma–Aldrich) at 37°C with aeration at 200 rpm. For growth on solid media, cells were plated on Luria Bertani Miller (LB) Agar media (Himedia) with incubation at 37°C for 2 days. The drug resistant strains, referred to as lab-selected mutants, were selected on increasing concentration of antibiotic containing plates (Padwal, 2016). The sigma factor knock-out mutants have been obtained from Prof. Robert Husson, at Boston Children’s Hospital. The oxidative stress mutants have been evolved, for the purpose of this study, in Middlebrook 7H9 media. For this, the cells were allowed to grow till optical density_600 nm_ (OD) of 0.5 in the presence of increasing concentrations of cumene hydroperoxide (CHP). Growth conditions for all the strains of *M. smegmatis* were similar to that of WT. *M. bovis* Bacillus Calmette–Guérin (BCG) strain has been obtained from All India Institute of Medical Sciences (Delhi) and was grown similarly in Middlebrook 7H9 media in absence of Tween-80. *M. bovis* BCG was handled in a Biosafety Level-2 facility.

To generate oxidative stress, the bacteria were subjected to the organic oxidant CHP with concentrations as indicated in the experiments. Both Rifampicin (RIF) and CHP, obtained from Himedia, were dissolved in Dimethyl sulfoxide (DMSO) for preparation of the stock and dilutions.

### Growth Kinetics and Viability Assay

The cells were grown till mid-log phase (OD of 0.5) in the absence of any stress and subsequently transferred to fresh media containing the stressor. The effect on the growth was monitored by measuring the OD of the cultures at defined time intervals. The viability of the cells was assessed by measuring the CFU/mL at the time intervals specified in the experiments. 10-fold serial dilutions ranging from 10^-1^ to 10^-6^ were plated on LB agar plates and incubated for 2 days. Viability was plotted as growth ratio obtained by normalizing by the initial CFU/mL of the culture.

### Determination of Minimum Inhibitory Concentration (MIC)

The MIC of RIF and CHP were screened using the checkerboard assay (Hsieh et al., 1993) and the disk diffusion assay (Bauer et al., 1966). For checkerboard assay, 0.5 OD cells (corresponding to approx. 10^7^ cells/mL) were diluted 10 times to attain 10^6^ cells/mL in Middlebrook 7H9 media from which 200 μL was aliquoted in a 96-well plate. Required concentrations of RIF and CHP were added to each well followed by 48 h incubation. Post treatment, the growth of the cells was estimated visually as well as by streaking 3 μL from each well onto a LB agar plate. The minimum concentration at which no growth was observed visually was considered as the MIC for the condition (Wiegand et al., 2008).

In the Disk Diffusion assay, 20 μL of 0.5 OD cells were streaked evenly on LB agar plate. Required amount of stressor was added on the sterile paper disk placed on the streaked cells and Zone of Inhibition (ZOI) around the disk was measured as per guidelines (Barry et al., 1979). The amount loaded on the disk is proportional to the MIC for a given ZOI, diffusion constant and time of diffusion (Kronvall, 1982). Both for CHP and RIF, the amount of drug loaded on the disk (μg) corresponding to a ZOI of 10 mm, was found to be equivalent to the MIC (μg/ml) determined by the checkerboard assay, as determined for the wild-type *M. smegmatis* strain. Thus, the content on disk (μg) that results in a ZOI of 10 mm, was considered to be equivalent to the MIC (μg/ml). Data throughout the manuscript has been presented in units of concentration.

### Fractional Inhibitory Concentration Index (FICI)

To calculate the degree of synergism between oxidative stress and RIF, the FICI was calculated as follows (Botelho, 2000):

$$FICI=\frac{\mathrm{MIC}\mathrm{of}\mathrm{drug}A\mathrm{in}\mathrm{combination}}{\mathrm{MIC}\mathrm{of}\mathrm{drug}A\mathrm{alone}}+\frac{\mathrm{MIC}\mathrm{of}\mathrm{drug}B\mathrm{in}\mathrm{combination}}{\mathrm{MIC}\mathrm{of}\mathrm{drug}B\mathrm{alone}}$$

For FICI of value ≤0.5, the effect of the combination was considered to be synergistic, whereas for values in the range of 0.5–4, the effect was considered to be additive and for values >4, the effect of was considered to be antagonistic (Odds, 2003).

### RIF Uptake

0.5 OD cells were centrifuged, concentrated to OD 5 and resuspended in Middlebrook 7H9 broth with 0.44% glycerol and 0.15% Tween-80 and the required concentration of RIF. The cells were further incubated for 1 h at 37°C with 200 rpm shaking. Uptake was measured by the difference in the RIF content of the media after 1 h. For analysis, 3 mL samples were removed at 0 and 1 h for each condition and were centrifuged at 8500 rpm for 10 min. Supernatant was filtered through 0.2 micron filter. The cell pellet was dried and the Dry Cell Weight (DCW) for each sample was measured. Standards were made in Middlebrook 7H9 broth and then filtered with 0.2 micron filter. RIF content was measured by running 10 μL of samples through 5 cm C18 column (Agilent make) in High Performance Liquid Chromatography (HPLC) Agilent 1260 infinity system. A 50:50 mixture of water acidified to pH 2.27 with o-phosphoric acid and acetonitrile was used as the buffer. Flow rate used was 1.2 mL/min and elute was measured with Ultraviolet (UV) detector at 333 nm.

### Estimation of Intracellular Reactive Oxygen Species (ROS)

Intracellular ROS was quantified using 2′,7′-Dichlorodihydrofluorescein diacetate (H_2_-DCFDA) dye (Keston and Brandt, 1965). Cells were grown till 0.5 OD and then subjected to the required conditions for either 1, 4, or 8 h, as indicated, at 37°C with aeration at 200 rpm. Post treatment, cells were centrifuged and then resuspended in PBS with 10 μM DCFDA. ROS converts the intracellular esterase-cleaved DCFDA into a fluorescent dye DCF which was then kinetically measured for 1 h (excitation at 488 nm and emission at 525 nm in Molecular Devices Spectramax M5) (Padwal et al., 2015). The end-point Relative Fluorescence Unit (RFU) is used for further calculations. The change in the viability of the cells after 8 h of treatment was taken into consideration by measuring the Colony Forming Unit (CFU)/mL. The normalized RFU at each time-point is calculated as follows:

$$\mathrm{Normalized}RFU=\frac{{\left(RFU/CFU\right)}_{\mathrm{condition}}}{{\left(RFU/CFU\right)}_{\mathrm{control}}}$$

### Expression Studies

Real time PCR was performed to study the expression level of *ahpC* and *ohr* after 15 min and 1 h of treatment with the conditions. RNA was purified using the trizol method (Sigma–Aldrich) followed by cDNA synthesis using Reverse Transcriptase (RT) Enzyme (Thermo Scientific). The reaction mixture for cDNA synthesis was as follows: 4 μg RNA template, 1U RT enzyme, 1X RT buffer, 2 mM dNTP’s, 0.2 μg Random primers, water to make the volume upto 20 μL. After completion of the reaction cycle, comprising of the following steps: 25°C for 10 min, 50°C for 1 h and a final extension of 70°C for 10 min, the mixture was diluted to obtain a final cDNA concentration of 100 ng/μL. The expression levels of both the genes were measured using 100 ng of cDNA template with 0.5 μM each of the specific primers (*ahpC* Forward primer: 5′CCACTGGAAGTGGACGAACT3′ *ahpC* Reverse primer: 5′TTCTGGCCCAAGGACTTCAC3′; *ohr* Forward primer: 5′ GGATGGTGACGTTGAGACG 3′ *ohr* Reverse primer: 5′ CCAAACACAACGTGAAGCTG 3′) with 1X SYBR green in Illumina Real-time PCR. The 2^-ΔΔC_T_^ method was used for analysis where 16s rRNA was used as the house keeping gene, followed by normalization with the untreated sample (*t* = 0 min).

### Estimation of Membrane Integrity

The extent of membrane permeabilization was assessed using 1-*N*-phenylnaphthylamine (NPN) dye (Helander and Mattila-Sandholm, 2000). Cells were grown till 0.5 OD and then subjected to the conditions for either 1, 4, or 8 h, as indicated, at 37°C with aeration at 200 rpm. Post treatment, samples were removed, centrifuged, and resuspended in 5 mM 4-(2-hydroxyethyl)-1-piperazineethanesulfonic acid (HEPES) buffer. 100 μL of sample and 100 μL of 50 μM NPN in HEPES was mixed in a 96-well plate. Fluorescence was measured immediately after addition (excitation at 355 nm and emission at 402 nm) (Padwal et al., 2015). CFU was also quantified for each sample. Here again, the RFU values were normalized as explained in section “Estimation of Intracellular Reactive Oxygen Species.”

The damage to the membrane after 8 h of treatment was visualized using Cryo Field Emission Gun-Scanning Electron Microscope (CRYO-FEG-SEM) technology (Merkal et al., 1973). The sampling and washing protocol was optimized in the lab. Briefly, samples were centrifuged and washed eight times with autoclaved water to remove any traces of media components and salts and diluted to a confluency of approximately 10^5^ cells/mL. The images were captured on CRYO-FEG-SEM (JSM-7600F) platform.

### Estimation of Membrane-Potential

Changes in the potential across the cell membrane were quantified by the 3,3′-Diethyloxacarbocyanine iodide (DiOC_2_) dye (Foss et al., 2012). Cells were grown till 0.5 OD and then subjected to conditions for 1 h. Post treatment samples were centrifuged and resuspended in PBS (without K^+^ ions) with 30 μM DiOC_2_. Samples were further incubated in dark for 1 h at 37°C with aeration at 200 rpm. Post incubation, 200 μL of sample was aliquoted in 96 well plate and the fluorescence was detected by excitation at 488 nm and emission at 520 and 620 nm for green and red fluorescence, respectively. Data was plotted as ratio of red/green fluorescence for each sample (Novo et al., 1999).

### Estimation of Lipid Peroxidation (LPO)

Lipid peroxides were quantified by ferrous oxidation-xylenol orange (FOX-II) assay. The Fox-II reagent was prepared with methanol (90%), H_2_SO_4_ (25 mM), FeSO_4_.7H_2_O (250 μM) and Xylenol orange (100 μM) (Master et al., 2002). Cells were grown till 0.5 OD and then subjected to conditions for 1 h. Post treatment, the samples were diluted 10 times in Middlebrook 7H9 media. 100 μL of the diluted sample was then mixed with 900 μL of the FOX-II reagent and incubated in dark for 30 min to allow the reaction to complete. 200 μL of the colored product was then measured spectrophotometrically at 560 nm. The absorbance values for the conditions were normalized by the absorbance value of the control.

### Statistical Analysis

All the experiments were performed with at least a minimum of three biological replicates. Statistical analysis for intergroup comparisons was carried out using single factor ANOVA, whenever more than two treatment groups were compared. Significance was established if *F* was greater than *F*_crit_ (at 5% significance level). Further, significance between two treatment groups was quantified using student’s *t*-test with equal variances. ^∗^*p* < 0.05, ^∗∗^*p* < 0.005, ns – not significant.

## Results

### Quantitative Determination of MIC of RIF and CHP

**FIGURE 1:**
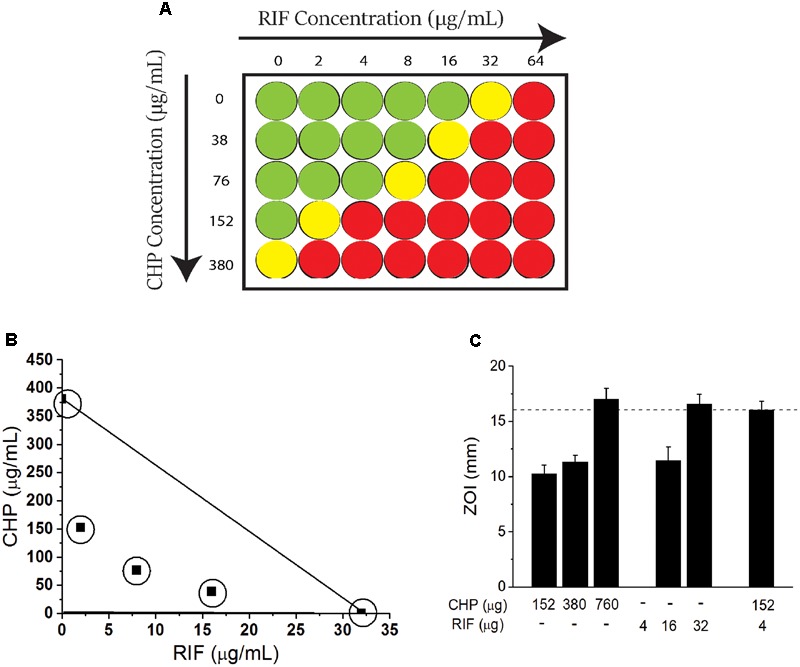
Effect of the combination of rifampicin (RIF) and Cumene Hydroperoxide (CHP) on minimum inhibitory concentration (MIC) of *Mycobacterium smegmatis* mc^2^155 cells. **(A)** Representation of checkerboard assay. Columns indicate RIF concentration. Rows indicate CHP concentration. Green represents growth, yellow represents growth inhibition and red represents lethality (determined as an absence of growth when culture is streaked on an agar plate). The concentration at which growth inhibition is observed is considered as the MIC. **(B)** Adaptation of Isobolographic analysis. The concentrations plotted here show growth inhibition as well as ZOI of 10 mm. The line of additivity connects the concentrations required by individual conditions. Combinations exhibiting growth inhibition were obtained below the line of additivity indicating the interaction of CHP and RIF to be synergistic **(C)** Comparison between the zones of inhibition of stationary phase culture of *M. smegmatis* mc^2^155 when subjected to CHP and RIF alone and in combination. Note that there is no zone of inhibition for 4 μg of RIF alone. Dotted line along the *Y*-axis represents an identical ZOI achieved (approximately 16 mm) by CHP and RIF individually as well as by the combination of the two.

A checkerboard assay (**Figure 1A**) was performed to screen the MIC of CHP and RIF alone and in combination. The MIC of RIF was identified as 32 μg/mL, whereas it was found to be 380 μg/mL (2.5 mM) for CHP. Further, the MIC of RIF could be decreased by many folds in combination with CHP. To illustrate, 152 μg/mL (1 mM) CHP plus 2 μg/ml RIF exhibit the same effect as 32 μg/mL of RIF alone, thus reducing RIF MIC by 16-folds in the *M. smegmatis* mc^2^155 strain. In the presence of 380 μg/mL CHP, complete lethality was observed at the RIF concentrations screened here.

### Combination of CHP and RIF Is Synergistic in Action against Both Rapidly-Growing and Stationary Phase Cells

To further quantitate the interaction between CHP and RIF, an adaptation of an isobolograph (Gessner, 1995) was plotted (**Figure 1B**). The line connecting 380 μg/mL CHP and 32 μg/mL RIF, denotes the line of additivity. If the combinatorial treatment resulting in growth inhibition is obtained with those concentrations of CHP and RIF which are below the line of additivity, then the interaction between the two would be considered as a synergistic interaction; for example the combinations of 76 μg/mL CHP + 8 μg/ml RIF and 38 μg/mL CHP + 16 μg/ml RIF, when plotted, demonstrate the interaction between RIF and CHP to be synergistic.

Further, the FICI was also estimated. The MIC values considered for FICI (refer Materials and Methods section) were as follows: CHP alone-380 μg/mL; CHP in combination-76 μg/mL; RIF alone-32 μg/ml and RIF in combination-8 μg/ml. With these values, the FICI obtained was 0.45. A FICI < 0.5 also indicated the interaction between CHP and RIF to be synergistic as per numerous guidelines (Odds, 2003; Johnson et al., 2004).

The synergistic interaction was also achieved on non-dividing, nutrient deprived stationary phase cells (day 8 culture) that were sensitive to both CHP and RIF, as compared to cells from the exponential phase. In the presence of a sub-inhibitory amount of 152 μg CHP, the required amount of RIF was reduced by eightfold to achieve the same zone of inhibition as RIF alone (**Figure 1C**).

### Robust Effect of CHP and RIF on Strains with Varying Sensitivities

Drug resistance is a rampant problem in the eradication of tuberculosis (Johnson et al., 2006), increasing the need for an improved shorter-regime treatment targeting resistance. We, therefore, screened the effect of a combination of RIF and CHP on *M. smegmatis* strains of different genetic backgrounds having variable susceptibility profile toward CHP and/or RIF. For example, for lab-selected antibiotic resistant strains, **Table 1** shows that the presence of 76 μg/mL CHP was able to synergistically decrease the MIC of RIF by four and eightfold in strains 1 and 2, respectively. A similar synergistic response was also observed for knock-out mutants of important extracytoplasmic function (ECF) sigma factors like SigH and SigE (Rodrigue et al., 2006). The effect of synergism was strongest in the *sigH* knock-out strain in which 76 μg/mL CHP was able to reduce the MIC of RIF from 32 to 2 μg/mL. Furthermore, CHP-resistant mutants were selected in the laboratory in the presence of increasing concentrations of CHP. A drastic 16-fold reduction in the MIC of RIF was observed in strain 2 in the presence of a sub-inhibitory concentration of 380 μg/mL CHP.

**Table 1:** Minimum inhibitory concentration (MIC) of rifampicin (RIF) and cumene hydroperoxide (CHP) individually and in combination on different strains of mycobacteria.

Effect of combinatorial treatment on different strains of mycobacteria					
	MIC for CHP (μg/mL)	MIC for RIF (μg/mL)	MIC for combination CHP + RIF μg/mL)	Fold change (RIF)	FICI
***M. smegmatis***					
Wild-type mc^2^155	380	32	76 + 8	4	0.45
**Antibiotic resistant strains (Lab-selected mutants)**					
Strain 1	380	64	76 + 16	4	0.45
Strain 2	380	256	76 + 32	8	0.33
**Antibiotic sensitive strains (sigma factor mutants)**					
Strain 1 (ΔsigB)	380	8	76 + 1	8	0.33
Strain 2 (ΔsigE)	380	16	76 + 4	4	0.45
Strain 3 (ΔsigH)	380	32	76 + 2	16	0.26
**Oxidative stress resistant strains (lab-selected mutants)**					
Strain 1	1140	8	380 + 2	4	0.58
Strain 2	1520	16	380 + 1	16	0.31
***M. bovis* BCG**					
BCG strain	152	0.03	38 + 0.0075	4	0.5

### Combination of CHP and RIF Is Synergistic on *M. bovis* BCG Strain

To ascertain that the beneficial effect of CHP can be translated to other species of mycobacteria, we screened the effect of the combination of CHP and RIF on the MIC of *M. bovis* BCG. Compared to *M. smegmatis*, the BCG strain was much more sensitive towards both CHP and RIF with growth inhibition observed at 152 μg/mL for CHP and 30 ng/mL for RIF (**Table 1**). The combination showed promising results with a very low concentration of CHP of 38 μg/mL, reducing the inhibitory concentration of RIF by fourfold giving a FICI of 0.5. These results on *M. bovis* BCG show increased likelihood of the synergistic effect to translate to pathogenic mycobacteria.

### Growth Kinetics in the Presence of CHP and RIF

**FIGURE 2:**
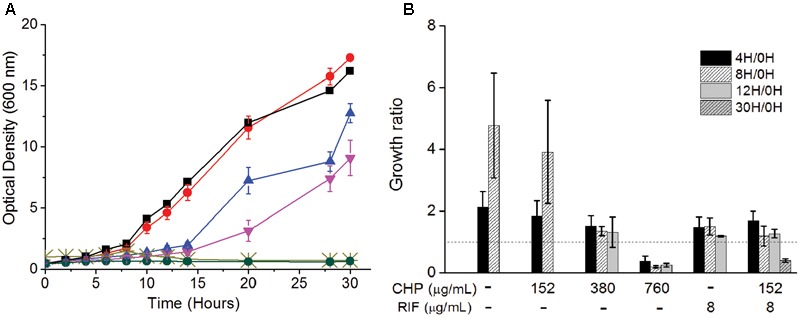
Effect on the **(A)** Growth kinetics of mc^2^155 cells on treatment with RIF (8 μg/mL) and CHP (152–760 μg/mL) alone and in combination. Here, black squares represent control, red circles represent 152 μg/mL CHP, blue triangles represent 8 μg/mL RIF, inverted purple triangles represent 380 μg/mL CHP, light green cross represents 760 μg/mL CHP and green circles represent the combination of 152 μg/mL CHP + 8 μg/mL RIF. **(B)** Viability (expressed in terms of growth ratio, defined as ratio of cfu/ml at indicated time-point to cfu/ml at 0 h) – in the presence of increasing concentrations of CHP (152, 380, and 760 μg/mL), RIF at 8 μg/mL and the combined stress of 152 μg/mL CHP plus 8 μg/mL RIF. Dotted line represents the growth ratio of 1 which indicates conservation of a constant viability. Growth ratio has not been plotted for cultures once they grow beyond 2.0 OD. For 760 μg/mL CHP, the growth ratio is almost 0 by 30 h of treatment.

The above data establishes the importance of a mild oxidative stress in inhibiting the growth of *M. smegmatis*. To understand the mechanism of action behind this inhibition, we determine the physiological behavior of the cells in the presence of synergistic combinations of CHP with RIF. Wild type *M. smegmatis* cells in mid-log phase (0.5 OD) were re-suspended in fresh media with either CHP alone, RIF alone or in combination. From the growth kinetics (**Figure 2A**) it was seen that 152 μg/mL CHP did not affect the growth of the cells. However, growth rate decreased in the presence of 380 μg/mL CHP, whereas 760 μg/mL CHP resulted in growth inhibition. In the presence of a sub-inhibitory concentration of RIF 8 μg/mL, cells exhibited a lag in the growth when initially exposed to the drug. But eventually, post 12 h of treatment the cells were able to resume growth. However, the combination of 152 μg/mL CHP + 8 μg/mL RIF exhibited complete growth inhibition. The growth kinetics was further supported by viability data, where a significant reduction in viability, of about 70%, was observed after 30 h of treatment with both CHP and RIF whereas the cells in the presence of RIF 8 μg/mL alone reach confluency (**Figure 2B**). Thus, the combination of 152 μg/mL CHP and 8 μg/mL of RIF was selected for further investigations on understanding the mechanism of synergy.

### Uptake of RIF Increases in the Presence of CHP

**FIGURE 3:**
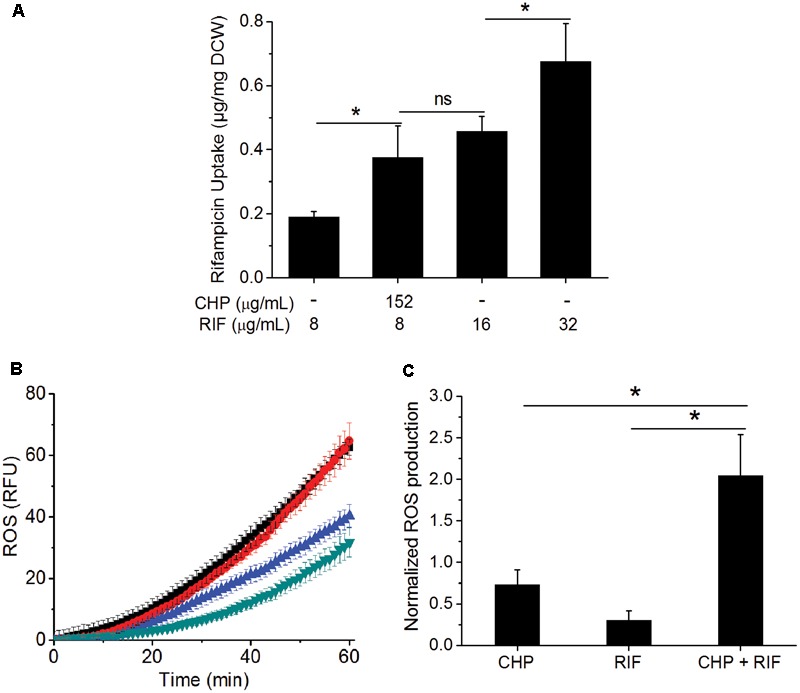
Effect of CHP on the drug uptake and ROS profile of the cells. **(A)** RIF uptake after 1 h of treatment with increasing concentrations of RIF (8, 16, and 32 μg/mL) compared to that of the uptake of 8 μg/mL RIF in the presence of 152 μg/mL CHP. **(B)** Kinetics of ROS production after 1 h of treatment with CHP (152 and 380 μg/mL) and RIF (8–64 μg/mL) individually and on the combined treatment of 152 μg/mL CHP plus 8 μg/mL RIF. Similar RFU values were obtained for Control and 152 μg/mL CHP and also for RIF concentrations from 8 to 64 μg/mL. Here, green triangles represent the profile of control as well as cells treated with 152 μg/mL CHP, blue triangles represent the average RFU under treatment with 8–64 μg/mL RIF, black triangles represent 380 μg/mL CHP and the red triangles indicate the ROS profile under combined treatment of 152 μg/mL CHP with 8 μg/mL RIF. **(C)** Normalized end point fluorescence after 8 h of treatment with 152 μg/mL CHP, 8 μg/mL RIF and its combination. ^∗^*p* < 0.05, ns – not significant.

One of the major barriers impeding drug activity is uptake of the drug by cells (Brennan and Nikaido, 1995). RIF uptake was measured in the single and the multi-stress condition to determine whether the inhibitory effect of the multi-stress condition could be attributed to higher drug uptake in the presence of CHP. **Figure 3A** shows that in the presence of 152 μg/mL CHP, uptake of RIF by the cells is significantly higher compared to drug alone. The uptake was similar to that observed when cells are subjected to an extracellular concentration of 16 μg/mL of RIF but not as high as corresponding to the MIC concentration of 32 μg/mL. Thus, the synergistic effect of CHP and RIF can only be partially attributed to the increased uptake of RIF in the multi-stress conditions.

### Increased Production of ROS in the Presence of Both RIF and CHP

As CHP leads to the production of ROS (Ayala et al., 2014), we next quantified this in single and multi-stress condition. **Figure 3B** shows that the ROS profile of 152 μg/mL CHP is similar to that of control. RIF treatment led to induction of ROS; although the phenomenon is independent of concentration, with 8–64 μg/mL RIF inducing similar ROS profile. Interestingly, 152 μg/mL CHP + 8 μg/mL RIF had significantly higher ROS compared to both the individual stresses, with the induction of ROS being similar to that observed in the presence of 380 μg/mL CHP. Furthermore, **Figure 3C** indicated the increased ROS production in the presence of multi-stress to be a long term effect, it being significantly higher than both the individual stresses, even after 8 h of treatment.

### Downregulation of *ahpC* and *ohr* in the Presence of RIF

Alkyl hydroperoxide reductase (AhpC) is one of the primary enzyme responsible for detoxification of ROS in the presence of organic peroxides (Hillas et al., 2000). In *M. tuberculosis*, knock-out mutants of *ahpC* gene have been observed to have increased sensitivity toward CHP with 50 μM CHP completely inhibiting the growth of the mutant in contrast to wild type cells (Springer et al., 2001). Similarly, downregulation of AhpC has been shown to increase the sensitivity of *M. smegmatis* cells toward CHP (Lee et al., 2014). In addition, *ohr*, has also been shown to be involved in the reduction of organic peroxides in *M. smegmatis* (Saikolappan et al., 2015). Garnica et al. (2017) have even demonstrated the importance of this protein in an infected model of *M. smegmatis*. Interestingly, *ohr* is absent in *M. bovis* BCG (Saikolappan et al., 2011) which can be a potential reason for the observed increased sensitivity of *M. bovis* BCG toward CHP.

**FIGURE 4:**
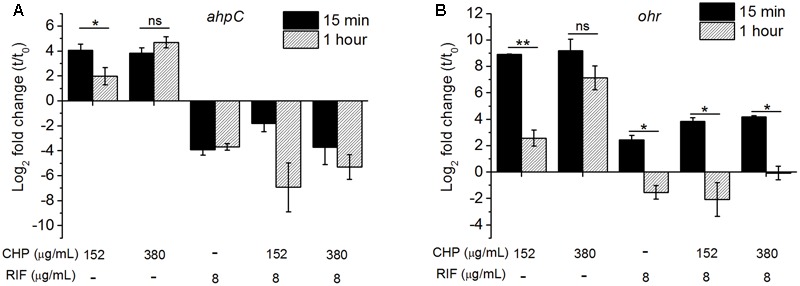
The relative expression level of **(A)** *ahpC*, **(B)** *ohr* after 15 min and 1 h of treatment with the stress conditions. The levels are normalized with 16s rRNA as the house-keeping gene, followed by normalization by 0 min (*t*_0_, untreated) sample. Significance between the expression ratio of 15 min and 1 h has been plotted here. Both *ahpC* and *ohr* in the presence of the combined treatment of CHP and RIF were significantly downregulated compared with that of CHP alone. ^∗^*p* < 0.05, ^∗∗^*p* < 0.005, ns – not significant.

We have quantified the *ahpC* and *ohr* mRNA levels in *M. smegmatis* on treatment with CHP as well as with RIF alone and in combination. The qRT analysis of *ahpC*, **Figure 4A**, showed transient upregulation of *ahpC* mRNA within 15 min of treatment with 152 μg/mL CHP. However, by 1 h of treatment, the mRNA levels were significantly lower. In cells treated with 380 μg/mL CHP, *ahpC* induction levels were maintained till at least 1 h post treatment. However, in the presence of sub-inhibitory concentration of RIF at 8 μg/mL, downregulation of the gene was observed. Interestingly, cells treated with 152 μg/mL CHP in combination with 8 μg/mL RIF also had repressed transcriptional levels of *ahpC.* Downregulation was also observed in cells treated with 380 μg/mL CHP and 8 μg/mL RIF. As shown in **Figure 4B**, *ohr* was upregulated in the presence of 152 and 380 μg/mL CHP alone. Similar to *ahpC*, mRNA levels remain upregulated under treatment with 380 μg/mL CHP. However, in the presence of RIF in combination with CHP, the *ohr* levels were significantly lower compared to that in CHP alone. Levels were further downregulated post 1 h of treatment.

### Loss of Membrane Integrity on Treatment with RIF and CHP

**FIGURE 5:**
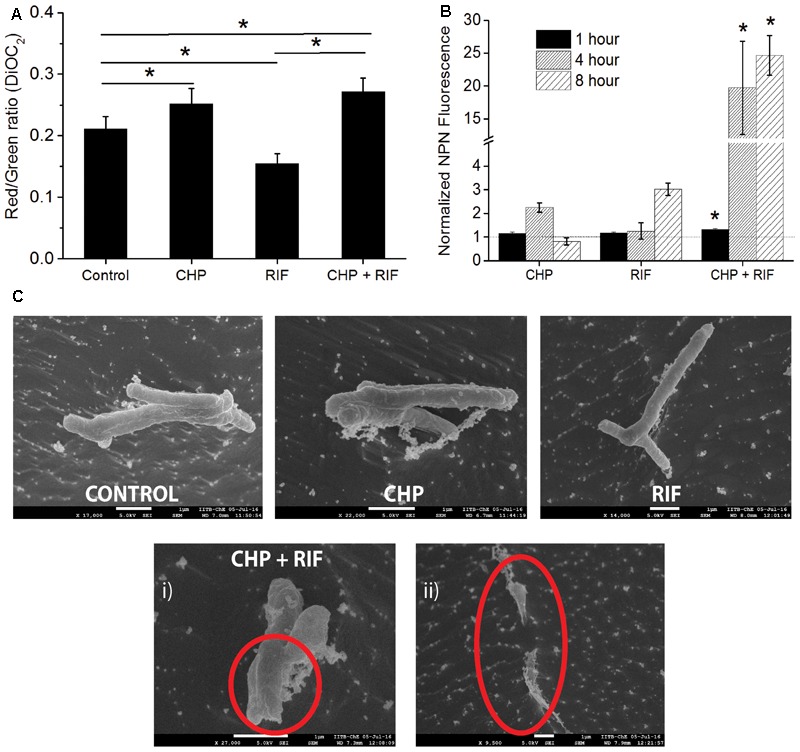
Estimating the effect of individual treatment of 152 μg/mL CHP and 8 μg/mL RIF and its combination on membrane integrity. **(A)** Membrane potential after 1 h of treatment denoted by the Red/Green ratio of DioC_2_ dye. Control represents the membrane potential of cells without exposure to any stressor. ^∗^*p* < 0.05. **(B)** Normalized membrane permeability after 1, 4, and 8 h of treatment. Dotted line represents the ratio of 1 indicating no significant membrane damage. ^∗^*p* < 0.05 with respect to both the individual treatments of CHP and RIF. **(C)** Comparison of cell morphology by CRYO-FEG-SEM imaging of cells after 8 h of treatment. Red circles highlight the damaged membrane observed after the combined treatment of CHP and RIF. Multiple frames were captured for the same. Individual images have been presented in the **Supplementary Figures S2–S6**.

Reactive oxygen species are known to cause LPO (Ayala et al., 2014) leading to loss of membrane integrity. In the multi-stress condition, since higher production of ROS was observed, increased damage to the membrane is expected. An immediate effect of membrane damage is the disruption of the membrane potential of the cells (Ohsuka et al., 1994). A healthy cell has a charge distribution across the membrane which is necessary for maintaining the physiological activities of the cells. Disturbance in this potential indicates a loss in the cellular activities which would eventually lead to cell death (Castillo et al., 2006). For instance, in mammalian cells, oxidative stress induced LPO increases the membrane rigidity which further leads to hyperpolarization of the membrane (Zavodnik et al., 1999; Jurkiewicz et al., 2012) whereas RIF treatment is known to induce membrane depolarization in *Escherichia coli* cells (Jammal et al., 2015). As seen in **Figure 5A**, treatment of *M. smegmatis* with CHP and RIF individually supports these earlier observations. Cells treated with 152 μg/mL CHP for 1 h are hyperpolarized with respect to untreated cells whereas in the presence of 8 μg/mL of RIF, cells were found to be depolarized. However, the combination of 152 μg/mL CHP and 8 μg/mL RIF caused hyperpolarization of the membrane, similar to that observed with 152 μg/mL CHP alone. This data suggests that production of ROS is the dominant factor in the multi-stress treatment.

An intact membrane is impermeable to hydrophobic molecules whereas damaged membrane has increased permeability toward these. Membrane permeability can be measured by the fluorescent probe, 1-N-phenylnaphthylamine (NPN) (Helander and Mattila-Sandholm, 2000), whose fluorescence increases in a hydrophobic environment. **Figure 5B** shows that in the presence of either 152 μg/mL CHP or 8 μg/mL RIF, when administered alone for 1 h, the normalized ratio of NPN fluorescence is approximately 1, indicating that these individual stress conditions do not cause any significant membrane damage to the bacteria. However, significantly high fluorescence is seen after 1 h of treatment with the multi-stress condition of 152 μg/mL CHP and 8 μg/mL RIF. The NPN ratio increases further after 4 and 8 h of treatment, indicating extensive damage to the membrane. This increase in membrane permeability can eventually lead to cell death. To additionally capture the membrane damage observed after 8 h of treatment, CRYO-FEG-SEM imaging was performed. The red circles in **Figure 5C** highlight the damaged membrane of cells in the multi-stress condition whereas cells treated with the single stress conditions have intact membranes and their cell morphology is similar to that of control.

### Not All Oxidative Stressors Are the Same

**FIGURE 6:**
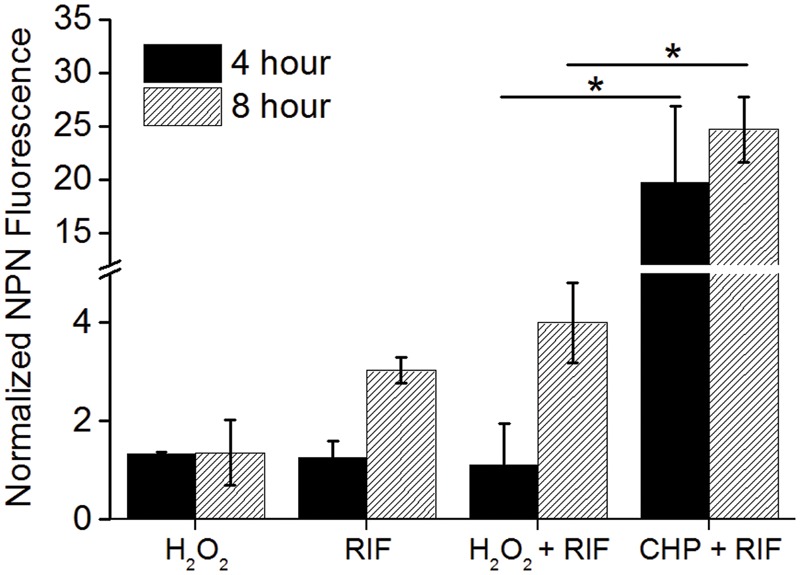
Normalized membrane permeability after 4 and 8 h of treatment with 85 μg/mL H_2_O_2_ and 8 μg/mL RIF individually and its combination. For comparison, permeabilization by 152 μg/mL CHP plus 8 μg/mL RIF is plotted. ^∗^*p* < 0.05.

While CHP was synergistic in action with RIF, the next important question that arises is will all oxidative stress molecules be synergistic with RIF? We, therefore, quantified the MIC’s of RIF in combination with tert-Butyl Hydroperoxide (t-BHP) and hydrogen peroxide (H_2_O_2_). t-BHP, similar to CHP, is known to induce LPO thereby triggering membrane damage (Kucera et al., 2014) whereas H_2_O_2_ can easily cross membranes and decompose inside a bacterial cell to form hydroxyl radical (Imlay, 2008). Similar to CHP, t-BHP also exhibited a synergistic action against *M. smegmatis* in combination with RIF (Supplementary Table S1). Interestingly, the combination of H_2_O_2_ and RIF resulted in an additive interaction, with the FICI being always greater than 0.5, against both wild-type and drug-resistant strains (**Table 2**). With CHP, membrane damage by the lipophilic alkoxy molecules leading to higher drug uptake had a significant role in growth inhibition. Consequently, we estimated the membrane damage in the combined treatment of H_2_O_2_ and RIF (**Figure 6**). However, in contrast to results obtained with CHP, membrane permeabilization in the presence of H_2_O_2_ and RIF, was similar to that of cells treated with the drug alone. Thus, not all ROS species may lead to a synergistic interaction with RIF.

**Table 2:** Minimum inhibitory concentration of RIF in combination with H_2_O_2_ as an oxidant against *M. smegmatis* wild-type and antibiotic resistant strains.

Effect of combinatorial treatment of H_2_O_2_ and RIF on *M. smegmatis* strains					
	**MIC for H_2_O_2_ (μg/mL)**	**MIC for RIF (μg/mL)**	**MIC for combination H_2_O_2_ + RIF (μg/mL)**	**Fold change (RIF)**	**FICI**

*M. smegmatis*					
Wild-type mc^2^155	170	32	85 + 8	4	0.75
**Antibiotic resistant strains (Lab-selected mutants)**					
Strain 1	170	64	85 + 8	8	0.63
Strain 2	170	256	85 + 64	4	0.75

### Lipid Peroxidation Is Important for Achieving Synergistic Response

**FIGURE 7:**
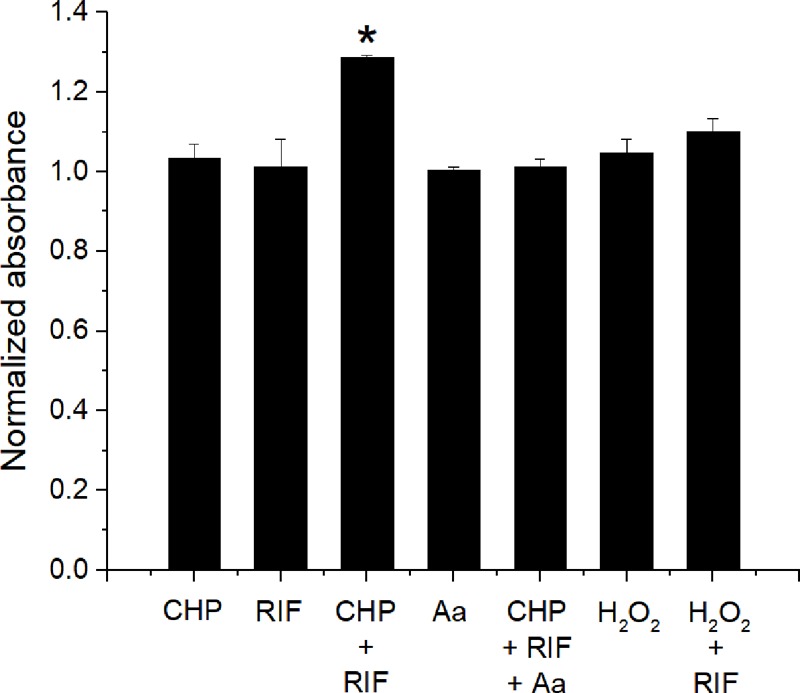
Estimation of production of lipid peroxides after 1 h of treatment with CHP (152 μg/mL) and RIF (8 μg/mL) alone and in combination. The peroxide levels are also estimated in the presence of 400 μg/mL of Ascorbic acid (Aa) applied alone and in combination with 152 μg/mL CHP plus 8 μg/mL RIF. Peroxides are also quantified in cells treated with H_2_O_2_ (85 μg/mL) alone and in combination with RIF (8 μg/mL). Normalized absorbance values represent the ratio of peroxides under treatment with respect to the control. ^∗^*p* < 0.05 with respect to all other conditions.

The interaction data of CHP, t-BHP, and H_2_O_2_ in combination with RIF, suggests that LPO is important to obtain a synergistic action. Therefore, the formation of lipid peroxides was quantified using the FOX- II assay (Master et al., 2002). *M. smegmatis* cells treated with CHP produce peroxides in a concentration dependent manner (**Supplementary Figure S1**). Further, as seen in **Figure 7**, after an hour of treatment, significantly higher peroxides were observed for the combined stress of 152 μg/mL CHP plus 8 μg/mL RIF, compared to the single stress conditions. Moreover, when CHP plus RIF is supplemented with sub-inhibitory concentration of 400 μg/mL of ascorbic acid (an antioxidant), the levels of the peroxides are reduced and similar to that of untreated cells. The presence of ascorbic acid was also able to suppress the inhibitory effect of CHP as seen by the loss of ZOI when applied in combination with 76 μg CHP plus 8 μg RIF and 152 μg CHP plus 8 μg RIF (Supplementary Table S1). In contrast, in cells treated with H_2_O_2_ in combination with RIF_,_ the amount of peroxides were significantly lower as compared to those treated with CHP + RIF. This data signifies the role of ROS species and its ability to induce LPO as one of the major reasons to achieve synergism.

## Discussion

Combination therapy with two or more drugs can help in improving treatment efficacy and in preventing antimicrobial resistance (Hill and Cowen, 2015). It is also one of the mechanisms to counteract resistant strains (Worthington and Melander, 2013). However, to design a combination with a synergistic interaction, it is important to understand the mechanism of action behind the interaction (Bollenbach, 2015). Here, we have explored in detail the mechanism behind the interaction of oxidative stress and a first line anti-tuberculosis drug, RIF. Previously, oxidative stress has been shown to be involved in the synergistic action of gentamicin and ampicillin (Barnes et al., 2005). In another example, RIF and Oligo-acyl-lysyl, while ineffective individually were found to be synergistic in action against Gram-negative bacteria when administered together (Jammal et al., 2015).

The choice for an oxidant in this study is an organic hydroperoxide. Organic peroxides form alkyl peroxyl radicals, in this case a cumoxyl/cumoyl peroxyl radical, in the presence of ferrous ions. These are chemically reactive molecules and cause stress by reacting with the hydrogen atoms of the cellular macromolecules (Akaike et al., 1992). Owing to the lipophilic property of these radicals, they tend to react with hydrogen atoms from the lipid bilayer of the membrane, instigating LPO and propagation, causing damage to the bacterial cell membrane and ultimately leading to loss of viability (Ayala et al., 2014).

The drug of interest, RIF, acts by intracellularly binding with RpoB, a β subunit of bacterial DNA-dependent RNA polymerase, leading to inhibition of the cellular transcription machinery and subsequently to cell death (Campbell et al., 2001; Malshetty et al., 2010). RIF has been clinically used against tuberculosis since as early as 1960s and is one of the most important drugs in killing the persister population of the infected bacteria (Mitchison, 2000). Even with the emergence of MDR and XDR strains, RIF and INH still remain the two most important drugs in the treatment regime of tuberculosis (Somasundaram et al., 2014). Moreover, RIF has been shown to have clinical therapeutic applications against other organisms like *M. leprae* (Gelber, 1998) and *Neisseria meningitides* (Deal and Sanders, 1969).

In this work we have shown that oxidative stress was able to drastically reduce the required inhibitory concentration of RIF. With checkerboard assay, a 16-fold reduction in the MIC of RIF was observed in combination with a sub-inhibitory concentration of CHP. Moreover, the FICI calculations and isobolographic analysis confirmed the interaction between the two to be synergistic. On exploring the physiological parameters, one of the reasons attributed for the synergism was higher drug uptake in cells in the presence of 152 μg/mL CHP. Intracellular uptake of RIF is directly proportional to the extracellular concentration. At inhibitory concentration of RIF of 32 μg/ml, we hypothesize that the corresponding intracellular concentration is sufficient to bind RpoB and inhibit cell growth. However, as the drug uptake in cells under the combined treatment of 152 μg/mL CHP and 8 μg/ml RIF is not as high as that achieved with 32 μg/ml RIF, it can be envisaged that the synergistic effect is a combination of multiple parameters and not just the effect of increased drug uptake.

We now summarize the proposed mechanism behind the three conditions of RIF 8 and 152 μg/mL CHP alone and the combination of the two. In the presence of a sub-inhibitory concentration of 8 μg/mL RIF alone, we hypothesize that the intracellular RIF binds with the RpoB protein. However, as the concentration of RIF is not high enough to completely inhibit transcription, the cells resume growth eventually. Intracellular uptake of RIF is proportional to the extracellular concentration, as observed in **Figure 3A**, therefore at inhibitory concentration of RIF of 32 μg/ml, RpoB is sufficiently inhibited to prevent cell growth.

When cells are treated with a sub-inhibitory concentration of 152 μg/mL CHP alone, there is no apparent effect on cell viability. Interestingly, the ROS and peroxides produced by cells treated with CHP for 1 h are similar to that of control. However, upregulation of the *ahpC* and *ohr* at 15 min post treatment, and further downregulation by 1 h suggests that the ROS may have been quenched by these enzymes, enabling the cells to grow normally. This is further supported by the sustained upregulation of both *ahpC* and *ohr* in cells treated with 380 μg/mL CHP, which also produce higher ROS compared to 152 μg/mL CHP. Additionally, the hyperpolarization of the membrane and the transient increase in permeability suggests that damage to the membrane in the presence of 152 μg/mL CHP alone is reversible. It is interesting to note that both *ahpC* and *ohr* are induced in response to organic peroxides, in *M. smegmatis*.

When cells are treated with a combination of RIF and CHP, increased RIF uptake has been observed, most likely due to the increased cell permeability in the presence of CHP. The higher intracellular RIF concentration potentially led to increased inhibition of RpoB, that further prevented the transcription of stress response genes as observed by the downregulation of *ahpC* and *ohr* in cells treated with RIF alone or in combination with CHP. While involvement of additional genes cannot be ruled out (Ung and Av-Gay, 2006; Saikolappan et al., 2011; Sao Emani et al., 2013), expression of AhpC and Ohr proteins is important to quench the ROS produced on treatment with CHP. We hypothesize that the absence of these proteins leads to production of additional ROS and peroxides as a consequence of the radical chain reaction. This subsequently intensifies the membrane damage, leading to heightened membrane permeabilization. The significant increase in membrane permeability in the presence of CHP and RIF together further supports the hypothesis that irreversible membrane damage has occurred. The consequent higher uptake of RIF induces clearance of the bacteria in due course of time.

Hydrogen peroxide, another oxidative stressor, exhibits only an additive effect in combination with RIF whereas t-BHP, which also generates an alkyl peroxyl radical like CHP, exhibited synergism. The common mechanism of action of all ROS species, including OH radical (OH^.^), Superoxide anion (O_2_^-^) and Hydrogen peroxide (H_2_O_2_), involves taking up of hydrogen atoms from cellular macromolecules. However, the distinct point of difference amongst these species and an alkyl peroxyl radical is the hydrophobic nature of the alkyl moiety. The alkyl groups being lipophilic, target lipid rich membranes as observed by quantification of higher lipid peroxides in case of the combination of CHP and RIF. In contrast, H_2_O_2_ and other ROS species are soluble in the cytosol making the cytoplasm their primary site of target (Hale et al., 2011). Microarray data of *Saccharomyces cerevisiae* (Sha et al., 2013) shows a distinct set of membrane genes differentially regulated by treatment with CHP which are not statistically different when treated with H_2_O_2_ indicating heightened damage to the membrane by CHP compared to H_2_O_2_. Thus, damage to membrane is key to the synergistic action of the combination of RIF with hydroperoxides. Therefore, for an enhanced therapeutic effect, we need to target a lipophilic reactive species in combination with RIF. Interestingly, RIF itself is also associated to be involved in the production of hydroxyl radicals (Piccaro et al., 2014). However, whether this has any role to play in the synergistic action in the presence of CHP needs further investigation.

## Conclusion

Our study presents a novel concept of proposing LPO causing oxidative agents, such as CHP, as adjuvants to the current regime of tuberculosis. Many nanoparticles such as magnesium oxide (He et al., 2016), cupric oxide (Applerot et al., 2012), zinc oxide (Xie et al., 2011), and silver nanoparticles (Quinteros et al., 2016) have been shown to cause oxidative stress, including LPO, leading to their antibacterial activity. Zinc oxide induced oxidative stress and membrane disintegration has been shown to have an enhanced effect on the clearance of *M. bovis* BCG (Pati et al., 2014). These nanoparticles can potentially be designed as antimicrobials that are selectively toxic toward the pathogen and not the host cells, leading to an enhanced synergy of RIF in the host. Thus, in this era of urgent requirement of better therapeutics, our work gives another direction for the development of antibacterial agents.

## Author Contributions

YP and SM conceived the study and designed the experiments. YP performed the experiments, analyzed the data and wrote the paper. SM analyzed the data and wrote the paper.

## Conflict of Interest Statement

The authors declare that the research was conducted in the absence of any commercial or financial relationships that could be construed as a potential conflict of interest.

## Supplementary Material

FIGURE S1 Standard plot of cumene hydroperoxide (CHP) in Middlebrook 7H9 for FOX-II assay.

FIGURE S2 Cryo Field Emission Gun-Scanning Electron Microscope (CRYO-FEG-SEM) image of control cells.

FIGURE S3 CRYO-FEG-SEM image of cells treated with 152 μug/mL of CHP for 8 h.

FIGURE S4 CRYO-FEG-SEM image of cells treated with 8 μg/mL of RIF for 8 h.

FIGURE S5 CRYO-FEG-SEM image of cells treated with 152 μg/mL of CHP plus 8 μg/mL of RIF for 8 h.

FIGURE S6 CRYO-FEG-SEM image of cells treated with 152 μg/mL of CHP plus 8 μg/mL of RIF for 8 h.
