# Supplementary material for: Synergistic Response of Rifampicin with Hydroperoxides on Mycobacterium: A Mechanistic Study
Source: Front Microbiol. 2017 Oct 31;8:2075. doi: 10.3389/fmicb.2017.02075 (PMC5671503; doi:10.3389/fmicb.2017.02075)
Supplement: Supplementary file 7 [file Table_1.docx]

Supplementary Material

Synergistic response of rifampicin with hydroperoxides on *Mycobacterium*: a mechanistic study

Yesha Patel, Sarika Mehra*

*** Correspondence:** Corresponding Author: sarika@che.iitb.ac.in

**Supplementary Table S1.** Zone of inhibition (ZOI) data on treatment of wild-type *M. smegmatis* with an organic oxidant t-BHP and an anti-oxidant ascorbic acid.

| **Conditions** | **ZOI (mm)** |
| --- | --- |
| **Effect of t-BHP on combination with RIF** | |
| 76 µg t-BHP | - |
| 380 µg t-BHP | 13 |
| 16 µg RIF | - |
| 64 µg RIF | 13 |
| 76 µg t-BHP + 16 µg RIF | 13 |
| **Effect of ascorbic acid on combination with CHP + RIF** | |
| 76 µg CHP | - |
| 152 µg CHP | - |
| 8 µg RIF | - |
| 76 µg CHP + 8 µg RIF | 10 |
| 152 µg CHP + 8 µg RIF | 12 |
| 400 µg Ascorbic acid | - |
| 1600 µg Ascorbic acid | - |
| 76 µg CHP + 8 µg RIF + 400 µg Ascorbic acid | - |
| 152 µg CHP + 8 µg RIF + 400 µg Ascorbic acid | - |
